# Analysis of Dietary Intake during Consecutive-Day Chemotherapy for Bone and Soft-Tissue Sarcomas

**DOI:** 10.3389/fnut.2017.00070

**Published:** 2018-01-22

**Authors:** Yuta Hori, Akio Sakamoto, Takashi Goto, Syouji Ando, Manato Yamashita, Masayo Shimomura, Takuji Uemura

**Affiliations:** ^1^Department of Pharmacy, National Hospital Organization, Kokura Medical Center, Kitakyushu, Japan; ^2^Department of Clinical Research Institute, National Hospital Organization, Kokura Medical Center, Kitakyushu, Japan; ^3^Department of Orthopaedic Surgery, Graduate School of Medicine, Kyoto University, Kyoto, Japan; ^4^Department of Nutrition Management, National Hospital Organization, Kokura Medical Center, Kitakyushu, Japan; ^5^Department of Pharmacy, National Hospital Organization, Oita Medical Center, Oita, Japan; ^6^Department of Pharmacy, Social Insurance Nakabaru Hospital, Fukuoka, Japan

**Keywords:** chemotherapy, nausea and vomiting, dietary, antiemetic, bone, soft-tissue, sarcoma

## Abstract

**Background:**

Bone and soft tissue sarcomas are commonly treated with consecutive-day chemotherapy regimens consisting of multiple anticancer agents. Chemotherapy-induced nausea and vomiting (CINV) is a serious adverse effect of these regimens and may result in decreased energy intake during chemotherapy. Decreased energy intake may lead to undernutrition and may cause adverse effects on patient quality of life and survival.

**Methods:**

Patients with bone and soft tissue sarcomas who received consecutive-day chemotherapy were retrospectively evaluated. CINV and dietary energy intake were assessed, as well as the occurrences of hiccups and constipation during chemotherapy.

**Results:**

A total of 13 patients, 10 males and 3 females, with a total 16 chemotherapy courses were included in the study. All patients received antiemetic prophylaxis. The CINV control rate, defined as no emesis and no rescue therapy, gradually decreased from chemotherapy day 1 (94%) to day 5 (75%). Four patients experienced emesis, two of whom had been treated with a cisplatin-containing regimen. Decreased dietary energy intake was possibly associated with CINV during chemotherapy. Anorexia was grade 2 except for one case of grade 3. The incidences of hiccups and constipation were high on days 3–5.

**Conclusion:**

Antiemetic prophylaxis treatment did not prevent emesis due to consecutive-day chemotherapy, especially with cisplatin-containing regimens, in patients with bone and soft-tissue tumors. Dietary energy intake decreased during chemotherapy, and this appeared to be associated with CINV. In addition, the incidence of hiccups and constipation increased during the course of consecutive-day chemotherapy regimens. Although these results are based on a small number of patients, it may be important to observe nutritional status during chemotherapy, as this may reflect a patient’s general condition. Nutritional counseling might be useful in supporting nutritional status in patients undergoing chemotherapy.

## Introduction

Chemotherapy-induced nausea and vomiting (CINV) is a nonhematologic toxicity associated with chemotherapy for malignant tumors (1). CINV is a collective term used to describe nausea, vomiting, or a combination of both symptoms, associated with chemotherapy. Although nausea and vomiting are related, they have distinct physiologic mechanisms (2, 3). Nausea is a subjective sensation of discomfort, typically associated with the epigastrium, which might result in vomiting. Because of its subjective nature, the sensation, location, duration, and intensity of nausea can vary (4, 5). CINV reduces patient quality of life and decreases treatment compliance. The degree of CINV is categorized by CINV frequency and is associated with the type, dose, and administration route of anticancer agents: low emetogenic risk, moderate emetogenic risk, and high emetogenic risk. Guidelines for the management of CINV recommend using a combination of dexamethasone and 5-HT_3_ receptor antagonists with or without Neurokinin 1 (NK1) receptor antagonists as antiemetic prophylaxis for moderate emetogenic risk chemotherapy regimens. Triple antiemetic prophylaxis including dexamethasone, 5-HT_3_ receptor antagonists, and NK1 receptor antagonists is recommended for high emetogenic risk chemotherapy regimens (3, 6).

Subclassification of CINV includes acute and delayed CINV. Acute CINV is generally considered to be nausea and/or vomiting that occurs within 24 h of chemotherapy administration. Delayed CINV is defined as nausea and/or vomiting that occurs after the first 24 h of chemotherapy administration (4, 7, 8). Different physiological mechanisms have been suggested to cause acute versus delayed CINV (9). The risk of acute or delayed CINV depends partly on the emetogenic potential of the anticancer agents (10). In consecutive-day regimens involving multiple anticancer agents, the risk of acute CINV overlaps with the risk of delayed CINV due to multiple days of therapy. Therefore, CINV may be persistent in patients receiving consecutive-day regimens involving multiple anticancer agents (11).

Soft tissue sarcomas are malignant tumors located in any of the mesodermal tissues of the extremities, trunk, retroperitoneum, or head and neck, and include more than 50 histologic subtypes (12). Cytotoxic chemotherapy using anticancer agents is the mainstay of treatment for advanced soft tissue sarcomas (13). The cytotoxic agents used to treat soft tissue sarcomas can be associated with significant adverse events, including pancytopenia, febrile neutropenia, nausea, alopecia, and fatigue (12). Consecutive-day chemotherapy regimens involving multiple anticancer agents are widely used to treat bone and soft tissue sarcomas (11). In many regimens, high doses of anticancer agents are administered and are thus frequently associated with CINV (11). In most cases, anthracyclines alone, such as doxorubicin (DXR), or in combination with other agents such as ifosfamide, are first-line treatment for advanced soft tissue sarcomas (12). During chemotherapy with DXR and ifosfamide for high-grade soft tissue sarcoma, 90% of patients experienced nausea despite antiemetic prophylaxis therapy. Severe nausea was seen in 26.4% of patients during preoperative chemotherapy and in 16.9% patients during postoperative chemotherapy (14). In a similar study, 85% of patients treated with prophylactic dexamethasone and 5-HT_3_ receptor antagonists suffered from nausea, and 70% of patients treated with dexamethasone, 5-HT_3_ receptor antagonists, and NK1 receptor antagonists (aprepitant) suffered from nausea (11).

Undernutrition or malnutrition is a serious clinical condition that causes adverse effects on patient quality of life and survival (15, 16). During cancer treatment, undernutrition is a risk factor for infectious complications and treatment intolerance (17). Moreover, undernutrition in patients with cancer is an adverse prognostic factor (18). CINV may result in malnutrition (19, 20). Patients experiencing CINV are susceptible to malnutrition due to the direct effects of nausea and vomiting (4). It has been suggested that nutritional status in patients with CINV should be actively monitored and managed to reduce the risk of malnutrition (4). Moreover, measurement of food intake is a proposed tool for evaluating abdominal symptoms and determining the need for rescue agents (21). In this study, the incidence of CINV was evaluated in patients undergoing consecutive-day chemotherapy for bone and soft-tissue sarcomas who received appropriate antiemetic prophylaxis. Dietary energy intake was also analyzed. Associated factors were analyzed, including the anticancer agents themselves.

## Materials and Methods

### Study Design

Patients diagnosed with bone and soft-tissue sarcomas and treated with consecutive-day chemotherapy from June 2012 to September 2013 at Kokura Medical Center were retrospectively reviewed. The incidence of CINV and the dietary energy intake were analyzed. All bone and soft-tissue tumors were stage III or IV, according to the American Joint Committee on Cancer stage classification. Antiemetic prophylaxis therapy was administered according to current guidelines (22–24). Patients receiving palliative chemotherapy were excluded. Patients younger than 15 years of age and older than 70 years of age were also excluded. The performance status of all patients was either 0 or 1. Successful CINV control during days 0–5 of chemotherapy was analyzed. Symptom control was defined as no emetic episodes and no use of rescue medications. Adverse events were defined by CTCAE ver. 4.0.

### Chemotherapy Regimens

Chemotherapeutic regimens were as follows: DXR regimen (30 mg/m^2^ DXR per day for 2 days), AI regimen (30 mg/m^2^ DXR per day for 2 days and 2 g/m^2^ ifosfamide per day for 5 days), AP regimen (30 mg/m^2^ DXR per day for 2 days and 120 mg/m^2^ CDDP), VDC regimen (1.5 mg/m^2^ (maximum dosage: 2.0 mg) vincristine, 30 mg/m^2^ DXR per day for 2 days, and 1,200 mg/m^2^ cyclophosphamide), and IE regimen (1.8 g/m^2^ ifosfamide per day for 5 days and 100 mg/m^2^ etoposide per day for 5 days). Each regimen was chosen according to the pathological diagnosis.

### Antiemetic Prophylaxis Therapy

Antiemetic prophylaxis therapy was based on the emetogenic risk of each chemotherapy regimen (3, 6, 25). The DXR and IE regimens were classified as moderate emetogenic risk. AI, AP, and VDC were classified as high emetogenic risk. A combination of 5-HT_3_ receptor antagonists (Granisetron) and dexamethasone was used for DXR. Triple antiemetic prophylaxis including NK1 receptor antagonists (Aprepitant), 5-HT_3_ receptor antagonists, and dexamethasone was used for IE, AI, AP, and VDC. Though the IE regimen is a moderate emetogenic risk regimen, triple antiemetic prophylaxis was used for the regimen (1).

### Prevention of CINV

Chemotherapy-induced nausea and vomiting prevention was defined as the absence of vomiting and the absence of rescue therapy for vomiting or nausea. Metoclopramide was used as rescue therapy.

### Calculation of Dietary Energy Intake

The amount of oral energy intake was calculated based on information gathered from medical records, nursing records, and the hospital food ordering system. Energy requirements were calculated using the Harris–Benedict equation, a formula that uses the basal metabolic rate and applies an activity factor to determine total daily energy expenditure.

### Constipation and Hiccups

The incidences of constipation and hiccups were evaluated based on information gathered from the medical and nursing records.

### Statistical Analysis

Fisher’s exact test was used to compare qualitative data including vomiting, chemotherapy regimens, hiccups, constipation, and patient background. The nonparametric Mann–Whitney *U*-test was used to compare quantitative data. The Kruskal–Wallis test was used, as appropriate, to compare dietary energy intake before starting chemotherapy and each day thereafter. A *P*-value less than 0.05 was considered statistically significant. All statistical analyses were performed using GraphPad Prism software, 5 for Windows Version 5.02 (GraphPad Software, Inc.).

### Institutional Review Board Statement

This study was reviewed and approved by the Ethics Committee of National Hospital Organization, Kokura Medical Center, Kitakyushu City, Japan.

### Informed Consent Statement

Patients were not required to give informed consent because the analysis used anonymous clinical data. On the home page of National Hospital Organization, Kokura Medical Center, all patients are alerted that anonymous data may be used for clinical studies.

## Results

### Patients

A total of 13 patients were enrolled in the study. Demographic data are presented in Table 1. The patients included 10 males and 3 females. The mean age was 39.7 ± 18.8 years old, ranging from 17 to 69 years with a median of 35.5 years. The mean body weight was 39.7 ± 18.8 kg, ranging from 40.4 to 120 kg with a median of 35.5 kg, and the mean body mass index was 27.2 kg/m^2^ (range: 19.1–40.5). The histological diagnoses and cancer subtypes were as follows: bone tumors included two osteosarcomas, one Ewing sarcoma, and one undifferentiated pleomorphic sarcoma; soft-tissue tumors included two malignant peripheral nerve sheath tumors, two synovial sarcomas, two Ewing sarcomas, one rhabdomyosarcoma (pleomorphic type), one leiomyosarcoma, and one– liposarcoma (dedifferentiated). Metastatic disease was seen in 6 of the 13 patients, and 8 out of 16 chemotherapy courses.

**Table 1:** Patient characteristics and chemotherapy courses.

Gender (*n*)	Male	10
	Female	3
Age (years)	Mean	39.7 ± 18.8
	Range	17–69
Body mass index (BMI)	Mean	39.7 ± 18.8
	Range	
Metastatic status	+	6
	−	7
History of chemotherapy	First line	13
	Second line	0
Previous number of chemotherapy courses	Average	1.25 (0–7)
	0	*n* = 10
	1	*n* = 3
	3–5	*n* = 3
Bone tumors (*n* = 4)	Osteosarcoma	2
	Ewing sarcoma	1
	Undifferentiated pleomorphic sarcoma	1
Soft-tissue tumors (*n* = 9)	Malignant peripheral nerve sheath tumor (MPNST)	2
	Synovial sarcoma	2
	Ewing sarcoma	2
	Rhabdomyosarcoma, pleomorphic type	1
	Leiomyosarcoma	1
	Liposarcoma, dedifferentiated	1
Metastasis	Positive	
	Negative	
Total (*n* = 13)		

### Chemotherapy and Antiemetic Prophylaxis Therapy

A total of 16 chemotherapy courses were reviewed. DXR chemotherapy was administered to six patients, AI chemotherapy was administered to three patients, AP chemotherapy was administered to two patients, VDC chemotherapy was administered to two patients, and IE chemotherapy was administered to three patients (Table 2). Patients receiving DXR therapy, a moderate emetogenic risk regimen, also received 3 mg 5-HT_3_ receptor antagonists as a single fixed intravenous dose that was administered 30 min before chemotherapy, and 6.6 mg dexamethasone. Patients receiving AI, AP, and VDC, high emetogenic risk regimens, and IE, a moderate emetogenic risk regimen, also received 3 mg 5-HT3 receptor antagonists and 6.6 mg dexamethasone as a single fixed intravenous dose that was administered 30 min before chemotherapy. Patients subsequently received 3 mg 5-HT_3_ receptor antagonists twice daily on days 1–4, and once daily on day 5. All patients received 125 mg NK1 receptor antagonists orally on the first day, and 80 mg/day thereafter. All 16 patients received first-line chemotherapy. The average number of previous chemotherapy courses was 1.25; 10 patients had received no prior chemotherapy, 3 patients had one prior course of chemotherapy, and 3 patients had three to five prior chemotherapy courses.

**Table 2:** Chemotherapy regimens for bone and soft-tissue sarcoma.

Regimens	Agents	Amounts	Day	Emetic risk	Antiemetics
DXR (*n* = 6)	DXR	30 mg/m^2^	Days 1–2	Moderate	Granisetron
					Dexamethasone
AI (*n* = 3)	DXR	30 mg/m^2^	Days 1–2	High	Granisetron
	IFO	2 g/m^2^	Days 1–5		Dexamethasone
					Aprepitant
AP (*n* = 2)	DXR	30 mg/m^2^	Days 1–2	High	Granisetron
	CDDP	120 mg/m^2^	Day 1		Dexamethasone
					Aprepitant
VDC (*n* = 2)	VCR	1.5 mg/m^2^	Day 1	High	Granisetron
	DXR	37.5 mg/m^2^	Days 1–2		Dexamethasone
	CPA	1,200 mg/m^2^	Day 1		Aprepitant
IE (*n* = 3)	IFO	1.8 g/m^2^	Days 1–5	Moderate	Granisetron
	VP-16	100 mg/m^2^	Days 1–5		Dexamethasone
					Aprepitant

*AI, doxorubicin and ifosfamide; AP, doxorubicin and cisplatin; CDDP, cisplatin; CPA, cyclophosphamide; DXR, doxorubicin; IE, fosfamide and etoposide; IFO, ifosfamide; VCR, vincristine; VDC, vincristine, doxorubicin and cyclophosphamide; VP-16, etoposide*.

### Frequency of CINV

**Figure 1:**
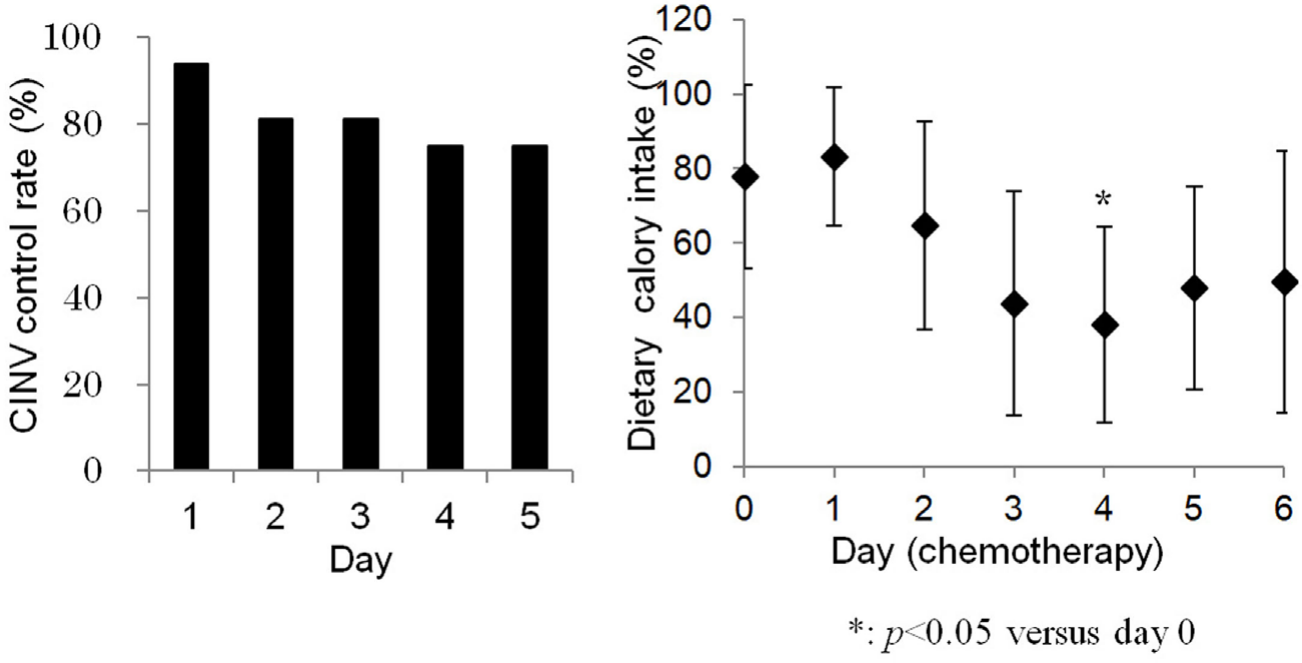
Chemotherapy-induced nausea and vomiting control gradually decreased during consecutive-day regimens (left). Dietary calory intake was significantly lower at day 4 compared to day 0 (right).

Vomiting occurred during four chemotherapy courses, in four different patients. All four chemotherapy courses included DXR. Furthermore, two contained cisplatin (AP regimen), one contained ifosfamide (AI regime), and one contained single-agent DXR. One vomiting episode was grade 3 (in a cisplatin-containing regimen), and the others were grade 1. Nausea was reported in the same four patients. One nausea episode was grade 3 (in a cisplatin-containing regimen), and the others were grade 2. Consequently, the successful prevention of CINV at day 1 was 93.8%. The successful prevention of CINV on days 2–5 was 81.3, 81.3, 75.0, and 75.0%, respectively. There was a tendency for CINV control rate to decrease over the course of treatment (Figure 1). No statistically significant correlations were observed between the occurrence of vomiting and patient gender or age, metastatic status, the emetic risk of treatment regimens, or history of chemotherapy (Table 3).

**Table 3:** Correlations between patient characteristics and the occurrence of vomiting, hiccups, and constipation.

		Vomit (*n* = 4)	No vomit (*n* = 12)	Hiccup (*n* = 3)	No hiccup (*n* = 13)	Const (*n* = 7)	No const (*n* = 9)
Age (years)	Mean	27.3	43.8	41.0	39.4	51.6^a^	30.4
		NS (*P* = 0.16)		NS (*P* = 0.69)		*P* = 0.026	
Gender	Male	4	8	3	9	4	8
	Female	0	4	0	4	3	1
		NS (*P* = 0.52)		NS (*P* = 0.53)		NS (*P* = 0.26)	
Metastatic state	+	1	7	2	6	3	5
	−	3	5	1	7	4	4
		NS (*P* = 0.569)		NS (*P* = 1)		NS (*P* = 1)	
Emetic risk	High	3	4	2	5	2	5
	Mod	1	8	1	8	5	4
		NS (*P* = 0.26)		NS (*P* = 0.55)		NS (*P* = 0.36)	
History of chemo	+	1	5	0	6	2	4
	−	3	7	3	7	5	5
		NS (*P* = 1)		NS (*P* = 0.25)		NS (*P* = 0.633)	
Cisplatin^b^	+	2	0	1	1	0	2
	−	2	12	2	12	7	7
		NS (*P* = 0.05)		NS (*P* = 0.35)		NS (*P* = 0.48)	

*Chemo, chemotherapy; Const, constipation; F, female; M, male; mod, moderate; NS, not significant*.

*^a^P < 0.05*.

*^b^Cisplatin, cisplatin-containing regimen*.

### Dietary Energy Intake

Dietary energy intake during days 0–6 of chemotherapy is shown in Figure 1. The average dietary energy intake on the day before chemotherapy initiation (day 0) was 77.9% of the expected caloric intake. Intake gradually decreased to 43.8% on day 3, 38.1% on day 4, and 47.9% on day 5. Decreased energy intake was associated with the occurrence of CINV (*P* < 0.05). Dietary energy intake was significantly lower on day 4, compared to day 0. Anorexia grades were less than 2, except in one patient with grade 3 anorexia (the same patient who experienced vomiting during a cisplatin-containing regimen).

### Incidences of Hiccups and Constipation

**Figure 2:**
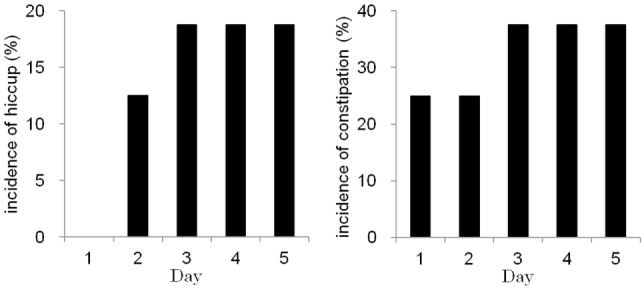
The incidences of hiccups (left) and constipation (right) were high on days 3–5 during consecutive-day chemotherapy.

Figure 2 shows the occurrences of constipation and hiccups during the chemotherapy courses. Hiccups occurred in 3 of the 16 chemotherapy courses. Hiccups were treated with hydroxyzine. The rate of hiccups on chemotherapy day 1 was 0%, whereas the rate on day 5 was 18.8%. The incidence of hiccups was not associated with demographic patient data, metastatic state, history of chemotherapy or cisplatin-containing regimens. Constipation occurred in 7 of the 16 chemotherapy courses. Constipation was treated with magnesium oxide, sennosides, or bisacodyl. Constipation tended to occur more frequently on days 3–5 than on days 1 and 2. The occurrence rate of constipation on chemotherapy day 1 was 25.0%, whereas the occurrence rate on day 5 was 37.5%. Constipation was significantly associated with older patient age; the mean age of patients with constipation was 51.6 years old, and the mean age of patients without constipation was 29.1 years old (*P* < 0.05) (Table 3).

## Discussion

The degree of CINV is based on CINV frequency and depends on the type and dose of anticancer agents. High emetogenic risk agents cause CINV in more than 90% of patients, moderate emetogenic risk agents cause CINV in 30–90% of patients, and low emetogenic risk agents cause CINV in less than 30% of patients (1). Consecutive-day regimens with multiple chemotherapy agents are commonly used to treat bone and soft-tissue sarcomas. In the current study, all chemotherapy regimens were either moderate or high emetogenic risk. Despite appropriate antiemetic prophylaxis therapy, successful prevention of CINV decreased from day 1 to day 5 of chemotherapy, and 4 out of 13 patients experienced vomiting. We observed no differences in CINV between patients with or without a prior history of chemotherapy. In a previous report of chemotherapy using DXR plus ifosfamide for soft tissue sarcomas, the incidence of severe nausea seemed to be lower in patients receiving postoperative chemotherapy (16.9%) than in those receiving preoperative chemotherapy (26.4%) (14). For patients receiving multicourse chemotherapy, early CINV control is important, because a history of poorly controlled CINV is a risk factor for future CINV (4, 26).

Evidence-based guidelines provide limited options for antiemetic therapy to prevent CINV in patients undergoing consecutive-day chemotherapy (27). It is unclear which antiemetic agents should be administered for consecutive-day regimens (1). Palonosetron has a longer half-life and greater 5-HT_3_ receptor binding affinity compared to other 5-HT_3_ receptor antagonists (28). Randomized controlled trials have shown that palonosetron leads to better control of delayed emesis and delayed nausea compared to other 5-HT_3_ receptor antagonists (29–32). However, consecutive-day granisetron was shown to be non-inferior to single-shot palonosetron for treating CINV in patients with bone and soft tissue sarcoma (1).

The risk of CINV is, in part, determined by the emetogenic potential of the chemotherapy regimen (4). CINV appears to be associated with cisplatin-containing chemotherapy regimens. The AP regimen (DXR plus cisplatin) is a combination of 120 mg/m^2^ cisplatin and 60 mg/m^2^ DXR, and is frequently used to treat sarcomas such as osteosarcoma. This regimen is classified as high emetic risk. The dosage of cisplatin is rather high compared to treatment regimens for other types of cancer (33). The AP regimen should be considered an extremely high emetic risk regimen (1). The chemotherapy regimens administered for bone and soft tissue sarcomas may require stronger antiemetic therapies. Metoclopramide has been shown to be effective for protracted nausea and vomiting during chemotherapy with advanced emetogenic risk agents (34). Another report suggested that the addition of olanzapine may be effective in CINV that is not controlled with triple antiemetic prophylaxis (35, 36).

Various chemotherapy-related symptoms can influence nutrition, including nausea, appetite loss, lack of energy, and taste changes (37). CINV are considered symptoms that influence nutrition and that can result in malnutrition (20, 38, 39). Malnutrition causes impairments of the immune system, performance status, muscle function, and quality of life (40). Moreover, cancer-induced malnutrition is associated with a decreased response to chemotherapy, more frequent complications, and severe toxicity (18). Malnutrition is also considered an independent risk factor for mortality (41, 42). Dietary interventions may help in the management of CINV (4). It has been suggested that patients with cancer should undergo nutritional counseling at the time of diagnosis and should be monitored throughout treatment (40). In the present study, dietary energy intake was assessed. No patient had severe anorexia. However, dietary energy intake decreased on day 4 of chemotherapy administration, which was related to the occurrence of CINV. One patient receiving a cisplatin-containing regimen had grade 3 anorexia. Antiemetic prophylaxis with triplet antiemetic combination therapy was not sufficient to control CINV or maintain dietary energy intake during chemotherapy for bone and soft tissue sarcoma.

The occurrences of hiccups and constipation were also analyzed in this study. Hiccups tended to occur more often on days 3–5 than on days 1 and 2. Cisplatin, dexamethasone, and NK1 receptor antagonists are known to cause hiccups (33). However, in our series, the incidence of hiccups was not associated with patient demographics or cisplatin-containing regimens. Constipation tended to occur more often on days 3–5 than on days 1 and 2. Elderly age was the only risk factor for constipation, consistent with a previous report (43). Anticancer agents like cisplatin and DXR cause diarrhea (44). On the other hand, 5-HT_3_ receptor antagonists can cause constipation (43). Therefore, the administration of 5-HT_3_ receptor antagonists during consecutive-day chemotherapy may have caused constipation in the current study. The exact features contributing to hiccups and constipation are unknown. However, the frequency of hiccups and constipation increased during consecutive-day chemotherapy, especially in elderly patients.

This study has several limitations. First, this is a retrospective study. Second, the number of cases is small, due to the rare nature of bone and soft-tissue tumors. Third, patients were observed for less than one week. The limited number of cases and the limited time course of this study cannot support a definitive conclusion. However, observing nutritional status, which may reflect general patient condition, may be important during consecutive-day chemotherapy for rare bone and soft-tissue sarcomas.

## Conclusion

Chemotherapy for bone and soft tissue tumors generally includes high doses of anticancer medications and consecutive-day regimens. We found that the incidences of hiccups and constipation increased during the course of consecutive-day chemotherapy. Furthermore, antiemetic prophylaxis therapy using a triple drug combination was not sufficient to control CINV, especially in patients receiving cisplatin-containing regimens. CINV was associated with decreased dietary energy intake during chemotherapy. Nutritional counseling may be helpful in supporting nutritional status during chemotherapy.

## Ethics Statement

This study was reviewed and approved by the Ethics Committee of National Hospital Organization, Kokura Medical Center, Kitakyushu city, Japan, where the research was performed.

## Author Contributions

YH, AS, TG, MS, and TU designed the study; YH, TG, SA, and MY performed the data collection and sample analysis; YH and TG analyzed the data; YH and AS wrote the article. AS had primary responsibility for the final content. All authors read, critically revised, and approved the final manuscript.

## Conflict of Interest Statement

The authors declare that the research was conducted in the absence of any commercial or financial relationships that could be construed as a potential conflict of interest. The reviewers YL, RN, and the handling editor declared their shared affiliation.
